# 

**Published:** 1846-04

**Authors:** 


### TO READERS AND CORRESPONDENTS.

We have received:---Lectures on the Nature and Treatment of Deformities. By R. W. Tamplin, F. R. C. L. E. In Select Medical Library. Edited by John Bell, M.D. (From the Publishers.)

Essay on the Philosophy of Vitality as contra-distinguished from Chemical and Mechanical Philosophy, and of the Modus Operandi of Remedial Agents. By Martyn Paine, M. D., Professor of the Institutes of Medicine and Materia Medica in the University of New York, &c. (From the Author.)

New Elements of Operative Surgery. By Alfred A. L. M. Velpeau. Translated by P. S. Townsend, M. D. Under the supervision of Dr. Mott. Vol. II.

Transactions of the Medical Society of the State of New York. Vol. VI, Part 3.

A defence of the Medical Profession of the United States. By Martyn Paine, A. M., M. D., Professor, &c. (From the Author.)

Anniversary Address to the New York Medical and Surgical Society, January 3d, 1846. By F. Campbell Stewart, M. D.

Catalogue of Jefferson Medical College; Session 1845-6.

J. & H. G. Langley's Medical Catalogue for 1846.

Boston Medical and Surgical Journal to April 15th. (In Exchange.)

New York Journal of Medicine and the Collateral Sciences to March, 1846. (In Exchange.)

The American Journal of Insanity, for April, 1846. (In Exchange.)

The American Journal and Library of Dental Science, for March, 1846. (In Exchange.)

The Medical News and Library, to April, 1846. (In Exchange.)

The Western Journal of Medicine and Surgery, to April, 1846. (In Exchange.)

The Journal of Health and Monthly Miscellany, for April, 1846. (In Exchange.)

The Medical Examiner, to January, 1846. (In Exchange.)

Southern Medical and Surgical Journal, to April, 1846. (In Exchange.)

The Bulletin of Medical Science, to April, 1846. (In Exchange.)

The Buffalo Medical Journal, to April, 1846. (In Exchange.)

The Western Lancet, to February, 1846. (In Exchange.)

The Missouri Medical and Surgical Journal, to March, 1846. (In Exchange.)

The St. Louis Medical and Surgical Journal, to April, 1846. (In Exchange.)

---

### CONTRIBUTORS.

Ira C. Bachus, M. D., Jackson, Michigan.
G. N. Fitch, M. D., Logansport, Indiana.
A. H. Howland, M. D., Ottawa, Illinois.
John F. Henry, M. D., Burlington, Iowa.
A. G. Henry, M. D., Pekin, Illinois.
Edward Lewis, M. D., Jackson, Michigan.
John McLean, M. D., Jackson, Michigan.
Daniel Stahl, M. D., Quincy, Illinois.

